# Counterparts

**Published:** 1887-06-11

**Authors:** 


### COUNTERPARTS.

BY A STUDENT OF EASTERN PHILOSOPHIES.

### CHAPTER I.

A MAN'S own eyes can take in objects more or less distinctly at a distance of thirty or forty miles, if he stand on the top of a sufficiently high mountain, and if the objects be big enough—such for example as churches, poplar-trees, and hillocks two or three hundred feet high. What he can't see clearly with his unassisted eye he enlarges four or five hundred times by means of his microscope, or takes to pieces and re-unites in his crucible. When he has climbed his mountain for big things, and adjusted his microscope and his crucible for small ones, and done what the Americans call his "level best" at these processes, he experiences a melancholy kind of satisfaction. He has measured the world which is his own portion of the Universe in a kind of way. He knows, more or less, its constitution and order. He has no means of obtaining verifiable knowledge of anything beyond this strictly limited range, and unverifiable knowledge is no knowledge at all, and therefore not worth having. And so he strives to make himself content.

Such is the condition to which modern Europeans are fast reducing themselves. Even their religion has now but little to do with infinity and immortality. It is no longer an angel with good news of an endless life, but a policeman with ten prohibitions in his pocket, and a stipendiary magistrate presiding in its holy of holies.

That kind of thing may do for chemical and physiological laboratories, and for the snuffy and passionless mummies who find heaven, not in their lover's eyes, but in the ecstatic discovery of a new Infusorian, or the demonstration of the cerebral ganglion of a hitherto undescribed mollusc. Well, gentlemen, you are welcome to the delights of your own occupation. No doubt the laboratory is to you very much more Elysian than Eden was to Adam in its brightest days. I do not envy you or interfere with you, or try with rude and inexperienced hands to dash to the ground the scientific images which you have so painfully and patiently set up. I leave you alone, and do my best to believe what you tell me.

But I cannot live on the mental and spiritual food you provide for me. My lungs won't breathe in your painfully heavy and unstimulating atmosphere. A kind of mental and moral asthma seizes upon me, and I am suffocated. Since you will give me neither food nor air that has anything of the Infinite in it, I have betaken me to other intellectual regions. The vast and dreamy philosophies of the East, with their boundless range of speculation and their unlimited vistas of hope, have given me new life.

Thinking and speculating thus, I can escape when I please from your world of grim and rigid necessity, and dwell in another sphere where what ought to be is, and where every being is in a condition of perfect beatitude.

On a certain day last summer it was my fortune to be in the Island of Bute. We had driven round to Kilchattan Bay, one of the loveliest spots among all the Western islands and highlands. The sea was placid, the sun declining a little to the west, the air softly moving, thousands of insects humming with gentle contentment. The rest of the party had wandered away, and I was as profoundly alone, so far as human beings were concerned, as Robinson Crusoe in the early days of his island life. And yet not alone. The whole world about me was full of the gentlest, sweetest, most soothing life. Here were no laboratories, no physiological experiments, no precise definitions, no miserable equations, no bars and fetters and steel handcuffs imprisoning the soul. For the time I was free. I laid the reins loose on the neck of the swift steed Imagination, and away she flew to the distant East, the flowery land of the rising sun and the mystic life. I slept and dreamed, and in my dreams all tangled skeins were unravelled, all injustice brought to an end. The dreaming thoughts finally concentrated themselves on my recent intellectual explorations in the wide fields of Eastern speculation. The struggles and trials of poetic souls in their strivings after their affinities took definite shape, and enacted before my imagination a drama of which the following is a more or less correct waking recollection.

Why Paul Travers had come to Westborough-on-Sea was for some time an unexplained mystery, even to himself. For seven years he had wandered in uncivilised parts of the globe, adopting the language, habits, and dress of the Oriental tribes among whom he had elected to live, and studying with such enthusiasm and admiration their peculiar temperament, character, and religious beliefs, that he had almost forgotten there was such a continent as Europe, and that Europeans were perhaps, in their own way, as well worthy of study as Asiatics. One day, however, while sitting in the abode of a devout but ragged Buddhist, who confidently expected the next stage of his existence to be Nirvana, striving like him to subdue his body and discipline his mind by meditation and fasting (not that Paul had any wish for or belief in that practical extinction which is the ultimate goal and heaven of the Buddhist), he was overcome by such an intense and irresistible longing to see the home of his childhood once more, that he rose, bade a hurried adieu to his ascetic friend, who wished him a speedy absorption into the Ocean of Being, and started the very next day for England, where he arrived in due time, clothed, shaved, and in his right mind.

The London season was at its height, and Paul, who was of good family and moderate means, and whose fame as a rather original-minded traveller had preceded him, found himself everywhere received with open arms, and plunged into a whirl of gaieties, frivolities, and so-called amusements, which effectually precluded any return to those long hours of meditation in which he had been wont to indulge nightly. As for fasting, it was out of the question: invitations to dinner were too frequent. Society had discovered that Paul Travers was a mesmerist and "thought-reader"; it accordingly treated him systematically as one of the lions of the season—a distinction which caused him much surprise and more dissatisfaction. He objected to put young ladies to sleep for the amusement of a curious or incredulous company, and absolutely refused to career blindfolded round a room in search of a pin which he did not want to find. At last he made up his mind to escape from the notoriety which had been thrust upon him, and about the middle of June disappeared quietly from London, leaving not a trace of his existence behind him.

Many were the conjectures as to what had become of him. Some insisted that he had gone back to the East to await his final absorption into the ocean of Infinite Being; others suggested that he had put himself into a trance and could not get out again; or that it was only his "astral body" which had visited England, or that his spirit was floating about invisible somewhere, while his body lay surrounded with celestial fire, like Zoroaster's in Mr. F. Marion Crawford's novel of that name.

Paul, in the meantime, was walking quietly along the Parade at Westborough-on-Sea, enjoying the fresh air and the exhilarating sense of freedom which he had missed so much in London, and admiring the bright faces and gay dresses of the girls who passed and repassed him, as they promenaded up and down to the strains of a not very harmonious brass band. It is true that most of these young ladies were dressed in the fashions of former years, modified more or less by provincial tastes and ideas; but Paul knew more of Persian than Parisian garments, and thought everything charming. Still, his fortnight's training in London life had not been entirely thrown away upon him. At all events, he very quickly distinguished the only London girl on the Parade from the others, and his attention became speedily riveted upon her. It was not that there was anything striking or ultra-fashionable in Edith Charteris's attire; her purse was too slender and her taste too artistic to allow of her indulging in elaborate or extravagant costumes; but her dress, simple as it was, had been arranged with an eye to harmony of line, colour, and detail, and was worn with that unmistakable, though indescribable, air which gives a town-bred woman so great an advantage over her country cousins, however charming or graceful the latter in their own way may be.

A subtle sense of inward satisfaction stole over Paul as his eyes rested on Edith Charteris's figure, and this feeling grew so intense as he drew nearer, that he almost feared to risk dispelling it by looking at her face. Her back was towards him as he approached, but the glimpse of her profile which he caught in passing reassured him so completely, that he very soon turned and came leisurely along the promenade to meet her. To anyone in a susceptible frame of mind, the moment was favourable for falling in love, for Edith's eyes were full of the sunset, and her thoughts were far away; she therefore looked extremely poetical, an expression which suited her *spirituel* type of beauty; and the enthusiastic Paul, glancing into the dark eyes which met his for a moment as he passed, saw and recognised his fate. Paul was not exactly a fatalist, but he was inclined to believe in an all-ruling destiny, shaping and adapting the actions of men to the end for which they were created. This end he somewhat vaguely considered to be spiritual perfection, unattainable indeed during this life, but more easily reached in the next state of existence if the soul could only find its counterpart and complement before the body died.

Regardless of the undeniable fact that in this vexatious world men occasionally fall in love with the wrong woman, or, in other words, with some other man's counterpart, Paul arrived rapidly at the conclusion that the hand of Destiny itself must have led him from Thibet to London, and thence to Westborough, for the sole purpose of meeting his second self in Edith Charteris. One look into the depths of her sun-suffused eyes convinced him of this, and he went home to bed in a state of exalted beatitude.

Paul was by no means a remarkable-looking man. He was tall and thin, with sharp features, and a complexion verging on the cadaverous. Since his return to England he had worn his hair close-cropped, and kept his face clean-shaven, and looked altogether so very like any other young English gentleman, that Edith would probably not have noticed him, had it not been for the yellow flame which suddenly leapt into his deep-set eyes, turning their blackness into translucent amber as his gaze encountered hers.

Edith experienced a slight shock, followed by a sudden quickening of the blood in her veins, for which she could by no means account. It could hardly have been love at first sight in her case, as she was already very much in love with a certain Mr. Archibald Bannerman, of whom she had been thinking while looking at the sunset, and to whom she was to be married at some indefinite period of time.

Mr. Archibald Bannerman was rich, handsome, and well-born; and Edith, who, like most girls who owe their charm to their expression rather than to any classical regularity of feature, had an extremely humble opinion of her own personal attractions, and whose father, a retired military officer, had for years lived persistently beyond his income, felt deeply grateful to Archie for his preference of herself. The fact that Lady Frances Bannerman, Archie's mother, had never called on her, or in any way acknowledged her existence, had caused her a good deal of uneasiness, and she had timidly proposed to Archie that the engagement should be broken off, as his relatives did not seem to approve of it; but the young man, though he seldom lost an opportunity of reminding his *fiancée* of the sacrifice he was making for her sake, refused to listen to such a suggestion, and talked a great deal of bluster about being his own master, and not a slave to be sold by his mother to the highest bidder; while Edith's parents were aghast at the bare idea of her allowing such a chance to slip through her fingers.

"I wish he had not been rich," said the much tried Edith, "then there would have been no trouble."

"No," said her mother, "for in that case I should never have permitted the engagement."

Edith made no reply, but she looked obstinate.

"'Gad!" exclaimed Captain Charteris, with a laugh, "it is a mercy the man has money, for no power in heaven or earth would have prevented Edith from marrying a beggar had she taken it into her head to do so."

Altogether Edith felt herself in an unpleasant position at this time, and was not sorry when her aunt invited her down to Westborough for a few weeks, even though in accepting the invitation she would separate herself from Archie Bannerman, who could not be expected to tear himself from London at the beginning of June.

It was a warm Saturday afternoon, about ten days after his arrival at Westborough, that Paul sallied forth from his hotel in order to sun himself on the Parade and worship his kindred soul afar off, according to his usual custom; but, after wandering up and down for nearly half-an-hour, looking so wistfully at every tall girl in white whom he met, that at least six young ladies went home with the firm conviction that he had escaped from a lunatic asylum, he was obliged to own to himself that his search was vain. He felt almost aggrieved at this faithless conduct on the part of his other self; and the sight of her aunt sitting in the midst of a bevy of garrulous old ladies, herself the most garrulous of them all, irritated him to such an unreasonable degree, that he left the Parade and betook himself to the beach, in savage humour indeed.

All this time the object of his search was sitting placidly on a rock not very far away, drinking in ozone and congratulating herself on having escaped for a little while from her good, kind, but somewhat tiresomely loquacious aunt. The shore in front of Westborough-on-Sea is, on the whole, sandy, but there is one part, scarcely a mile from the north end of the esplanade, where flat rocks run far out into the sea, and where, when the tide is low, countless little boys may be seen fishing for crab and other unlucky crustaceans. On this particular Saturday afternoon, however, no little boys were there, for the tide was coming in, and the sea was gaining on the land with great quickness and quietness With a courage born of ignorance, Edith had seated herself near the edge of the water, on what had then been part of the mainland, but was now a rapidly diminishing island, and had speedily been lulled into a day-dream by the monotonous dash of the waves, as they crept closer and closer to her feet, wound themselves round the rock on which she was sitting, and spread themselves swiftly out between her and the distant shore.

"Is it your deliberate intention, young lady, to seek an early and watery grave?"

Edith started from her dreams and looked round. Paul was standing behind her with his legs bare to the knee; a broad expanse of water, through which he had evidently waded, separated her from the beach. Edith rose to her feet with an exclamation of alarm.

"I saw you from the shore," explained Paul, trying to speak in a matter-of-fact tone, "and thought I had better come across and fetch you without any preliminary shouting, which would only have alarmed you needlessly. You must allow me to carry you back."

"Oh, thank you—you are very kind—but I could not think of such a thing," answered Edith, hastily. "I can easily wade across if you will help me a little."

"As you please," said Paul, with a twitch of his lips; "but I must warn you that the water reached to my knees in some places as I came over, and it has not become shallower since."

"Oh!" said Edith, blankly.

"Come," said Paul persuasively, "there is no time to lose; you had better let me carry you."

"Very well," answered Edith, looking resigned; "but I am not very light."

Paul laughed and took her in his arms. Edith, whose ideal of manly strength was the ruddy-cheeked, broad-shouldered, portly British squire, as typified in Archie Bannerman, was surprised at the nervous power with which this thin, sparely-built stranger held her. She felt as safe and, as far as physical sensations were concerned, as comfortable in his arms as in an easy-chair at home. This happy state of security, however, soon received a shock, for Paul, having fallen upon stony ground, suddenly made a false step, which very nearly sent him and his fair burden headlong into the water. He was erect again in an instant, but Edith in her panic had clasped him round the neck and almost strangled him. Excepting this little mishap no other incident occurred till the shore was reached.

(*To be continued.*)

